# Use of virtual slide system for quick frozen intra-operative telepathology diagnosis in Kyoto, Japan

**DOI:** 10.1186/1746-1596-3-S1-S6

**Published:** 2008-07-15

**Authors:** Yasunari Tsuchihashi, Terumasa Takamatsu, Yukimasa Hashimoto, Tooru Takashima, Kooji Nakano, Setsuya Fujita

**Affiliations:** 1 Louis Pasteur Centre for Medical Research, 12-7 Taniguchi Kakinouchi, Ukyo, Kyoto, 616-8012, Japan; 2 CLARO Inc., 56-10, Honcho, Hirosaki, Aomori, 036-8203, Japan; 3 Yamashiro Public Hospital, 74-1, Kizu-Ikeda, Kizu, Kyoto, Japan

## Abstract

We started to use virtual slide (VS) and virtual microscopy (VM) systems for quick frozen intra-operative telepathology diagnosis in Kyoto, Japan. In the system we used a digital slide scanner, VASSALO by CLARO Inc., and a broadband optic fibre provided by NTT West Japan Inc. with the best effort capacity of 100 Mbps. The client is the pathology laboratory of Yamashiro Public hospital, one of the local centre hospitals located in the south of Kyoto Prefecture, where a fulltime pathologist is not present. The client is connected by VPN to the telepathology centre of our institute located in central Kyoto. As a result of the recent 15 test cases of VS telepathology diagnosis, including cases judging negative or positive surgical margins, we could estimate the usefulness of VS in intra-operative remote diagnosis. The time required for the frozen section VS file making was found to be around 10 min when we use ×10 objective and if the maximal dimension of the frozen sample is less than 20 mm. Good correct focus of VS images was attained in all cases and all the fields of each tissue specimen. Up to now the capacity of best effort B-band appears to be sufficient to attain diagnosis on time in intra-operation. Telepathology diagnosis was achieved within 5 minutes in most cases using VS viewer provided by CLARO Inc. The VS telepathology system was found to be superior to the conventional still image telepathology system using a robotic microscope since in the former we can observe much greater image information than in the latter in a certain limited time of intra-operation and in the much more efficient ways. In the near future VS telepathology will replace conventional still image telepathology with a robotic microscope even in quick frozen intra-operative diagnosis.

## Introduction

The practical applications of virtual slide (VS) or virtual microscopy (VM) system have been confined to such fields as education, conferences or consultations, where we are allowed enough time to digitize whole a slide specimen and make up a VS before starting observation. However through the recent rapid technical progresses of digital slide scanner for VS, the time required for making up a VS file is greatly shortened to the order of 10 min or so, so that we can now utilized VS and VM for quick frozen intra-operative telepathology diagnosis. For the last 15 years our telepathology project in Kyoto, Japan has been conducted using a static image telepathology system equipped with a remote controllable robotic microscope [1]. The system can now be called "conventional", in contrast to the "novel", VS telepathology system. In this paper we report our first trial of applying VS telepathology system to quick frozen intra-operative telepathology diagnosis. Merits of the use of VS system will be discussed.

## Materials and methods

We used a highly elaborate digital slide scanner, VASSALO, by CLARO Inc., Aomori, Japan [2] in a client pathology laboratory of Yamashiro Public Hospital in the south of Kyoto, Japan, where fulltime pathologist is not present by a well-trained cytotechnician is present. The client terminal is connected to our telepathology diagnostic centre in central Kyoto by a broadband optic fibre provided commercially by NTT-Wes Inc., Japan that has a best-effort capacity of 100 Mbps. The broadband was used solely for image data transmission and the client terminal and our telepathology diagnostic centre formed a VPN, called a B-Flets group. The time required for VS making of each frozen section was measured. At the same time, the time and accuracy of the remote observation of the VS by a VS viewer provided by CLARO Inc. were checked and recorded. Usefulness of VS telepathology diagnosis is estimated in terms of time required, VS quality, and of accuracy of diagnosis. The VS telepathology diagnosis was compared to those by conventional telepathology system by making diagnoses using VS and conventional systems successively for a same frozen section slide specimen.

## Results

In the last 6 months we conducted 15 test cases of quick frozen VS diagnosis. The time required for VS making was around 10 min if we use ×10 objective and if the specimen measures less than 20 mm in the maximal dimension. VS making was successful for all the tissue section cases. i.e., good focus of VS images was achieved in all the frozen section cases and in all the tissue fields of each case. Telepathology observation and diagnosis by VS viewer were achieved within 5 min and all the telepathology diagnoses were correct. The best effort type B-band optic fibre maintained sufficient capacity to attain remote diagnosis on line and on time.

## Discussion

Owing to the rapid and accurate digitalization of the whole of a glass slide specimen by VASSALO it was now clear that we can successfully use VS telepathology system even for quick frozen intra-operative telepathology diagnosis where time is quite limited for a technician and a remote pathologist. Telepathology diagnosis was easier and more comfortable in the novel VS telepathology system than in the conventional telepathology system since we can obtain desired images easier in VS system than in the conventional system. We can attain much greater image information processing in the VS system than in the conventional telepathology system in a certain limited time.

## Conclusion

VS system was used for quick-frozen intra-operative telepathology diagnosis in Kyoto, Japan. The VS system was found to attain correct diagnosis on time, i.e., VS making within 10 min and VS telepathology observations/diagnoses within the next 5 min. The novel VS telepathology system was found to be superior to the conventional still-image system in terms of time required for diagnosis and of ease and accuracy of the diagnosis. The use of VS system will be expanded from education to actual diagnosis including quick frozen intra-operative diagnosis. In the near future the conventional telepathology system to support regional medicine will be replaced by VS telepathology system.
